# Childhood musculoskeletal impairment in Malawi from traumatic and non-traumatic causes: a population- based assessment using the key informant method

**DOI:** 10.1186/s12891-021-04942-x

**Published:** 2021-12-21

**Authors:** Cortland L. Linder, Oluwarantimi Atijosan-Ayodele, Linda Chokotho, Wakisa Mulwafu, Myroslava Tataryn, Sarah Polack, Hannah Kuper, Hemant Pandit, Chris Lavy

**Affiliations:** 1 Royal London Hospital, Whitechapel Rd, Whitechapel, London, E1 1FR UK; 2 Maidstone and Tunbridge Wells NHS Trust, Tonbridge Rd, Royal Tunbridge Wells, Tunbridge Wells, TN2 4QJ UK; 3 Department of Surgery, College of Medicine, University of Malawi, Blantyre, Blantyre, Malawi; 4 International Centre for Evidence in Disability, London School of Hygiene & Tropical Medicine, London, WC1E 7HT UK; 5 Leeds Teaching Hospitals NHS Trust, Leeds, UK; 6 Nuffield Department of Orthopaedics Rheumatology and Musculoskeletal Science, Oxford University, Oxford, UK

**Keywords:** Disability, musculoskeletal impairment, trauma, children, key informant method, Malawi, service provision

## Abstract

**Background:**

Musculoskeletal impairment (MSI) in children is an under-recognised public health challenge. Although preventable, road injuries and other traumas continue to cause significant impairments to children worldwide. The study aimed to use the Key Informant Method (KIM) to assess prevalence and causes of MSI in children in two districts in Malawi, estimating the associated need for services provision, with a focus on traumatic aetiology.

**Methods:**

The KIM was conducted in the districts of Thyolo (Southern Malawi) and Ntcheu (Central Malawi) in 2013. Five hundred key informants were trained to identify children who may have one of a range of MSI. The identified children were referred to a screening camp where they were examined by medical experts with standardised assessment protocols for diagnosing each form of impairment.

**Results:**

15,000 children were referred to screening camps. 7220 children were assessed (response rate 48%) for an impairment of whom 15.2% (1094) had an MSI. 13% of children developed MSI from trauma, while 54% had a neurological aetiology. For MSI of traumatic origin the most common body part affected was the elbow. Less than half of children with MSI (44.4%) were enrolled in school and none of these children attended schools with resources for disability. More than half of children with MSI (60%) had not received required services and 64% required further physical therapy.

**Conclusions:**

The KIM method was used to identify a high prevalence of MSI among children in two districts of Malawi and estimates an unmet need for dedicated MSI services.

## Introduction

Musculoskeletal impairment (MSI) in children presents a significant public health challenge [1]. The World Health Organisation (WHO) estimates that 5% of children have moderate or severe disabilities worldwide, with higher prevalence in lower and middle income countries (LMICs) [2, 3]. With a worldwide reduction in child mortality, public health focus is shifting to improving quality of life for children [2]. The United Nation (UN) Sustainable Development Goals include the improvement of physical and psychosocial health and education of children less than 5 years old [4]. Furthermore the UN Conventions on the Rights of the Child and on the Rights of Persons with Disabilities together promote the rights of children with disabilities [5]. Children with disabilities are more likely to miss school and develop other serious illness, demonstrating a significant loss of potential and violation of rights [3]. Reliable surveillance of disability in children is vital to improving health services worldwide to provide appropriate care for these children. However, gathering data on the prevalence and distribution of impairment types and causes is difficult and expensive [2, 6]. To date, studies have used differing tools and methodology, producing data that are difficult to compare between countries [6].

Malawi has a population of 17.6 million people [7]. It is an extremely impoverished country with one of the lowest life expectancies worldwide [7]. In 2008 a Malawi Housing and Population Census estimated that the prevalence of all disabilities in children was 2.4%, although the census was limited by its lack of child-specific methodology and consequently the figure is likely to be an underestimate [8]. Injuries from trauma are common in Malawi, where an estimated 14% of deaths for 5-24 year olds are due to unintentional injury, most commonly transport-related [9]. For every death, road injuries are likely to leave several more with long term impairments [10]. Injuries are an important cause of long-term disability from a public health perspective as they are preventable, although trauma services are disproportionately underfunded in LMICs [11]. Malawi has no formal pre-hospital care service and 84% of the population live in rural areas with limited access to hospital care [9]. With the rapid rise in road injuries and growth in car ownership in Malawi, the magnitude of MSI from trauma is likely to increase if there is no intervention [12].

The Key Informant Method (KIM) is an approach designed to estimate prevalence and causes of childhood impairments in LMICs [13–15]. This method trains volunteers to be key informants (KI) within their local area to identify children with likely impairments. These children are referred to a screening camp, where they are examined by medical experts to confirm the diagnosis and recommend necessary treatments. This methodology is cheaper and quicker than population-based surveys, fosters engagement with communities and advises follow-up for affected children. In 2013 a survey was undertaken using the KIM in two districts in Malawi [15] to estimate the prevalence of intellectual, visual, hearing and musculoskeletal impairments and epilepsy. This estimated a prevalence of moderate/severe MSI of 5.8-7.6 cases per 1000 in children aged less than 18 years old.

This paper will explore the MSI data in further detail, including anatomical distribution, aetiology, severity and treatment required or received. It will focus on the prevalence of trauma in the paediatric population of Malawi in order to aid mapping of trauma prevention and services.

## Methods

### Ethics

Ethical approval for this study was granted from the London School of Hygiene and Tropical Medicine and the College of Medicine Research Ethics Committee, Malawi. Each child was accompanied by their parent/guardian. Participants and parents/guardians were informed about the study aims and methodology and consent was obtained with signature or thumbprint from the parent/guardian of all participants. On diagnosis of impairment, each participant was referred to the relevant service. All methods performed in this study were conducted in accordance with the relevant guidelines and regulations.

### Study Definitions

The International Classification of Functioning, Disability and Health (ICF) have created a framework to describe disability that includes health conditions, environmental and personal factors, participation, activities and impairments. This study assessed MSI, defined by the ICF as “…a lack of normal structure or function, or an increase in pain or discomfort in the integument, muscles, bone or joints of the body of an individual, that has lasted at least 1 month and which limits function of the musculoskeletal system…” [16]. Due to limited services and wide-ranging barriers in Malawi, children with MSI are more likely to be restricted in participation or activities but these were not assessed by the study. There are 8,895,000 estimated children under 18 years old in Malawi according to the 2008 census [8].

### Location

The study was undertaken from April to November 2013. Thyolo (Southern Malawi) and Ntcheu (Central Malawi) districts were selected for this study due to availability and proximity of healthcare services. The districts chosen were thought to approximate the socio-economics of the average rural district in their region [17]. The total number of children <18 years in these districts was estimated to be 338,000, using updated figures from the 2008 census.

### Key Informants

In each district, 250 volunteers from the communities were selected as Key Informants (KI). Selection was organised by Health Surveillance Assistants (HSAs), members of the community involved in public health surveillance, and District Health and Environmental Officers. The KIs in each area were supervised by HSAs, who also determined the location of screening camps. The volunteers were trained for one day in groups of 20 by the project co-ordinator using a training manual, which had details about the relevant impairments and the methodology of the screening. Following this, the KIs had up to 6 weeks to identify and record children in their community who may have an impairment. Identified children and their guardians were invited to attend local screening camps. All KIs and HSAs were reimbursed for their transport and work.

### Screening camps

There were 33 screening camps in total, each with three orthopaedic clinicians who rotated between clinical work and screening camps. The clinicians received one day of training on the study protocols, which was monitored by field supervisors.

Children and their guardians underwent an initial screening questionnaire. To assess for musculoskeletal impairment, participants completed the following 7-question screen [15]

1. Is any part of your body missing or misshapen?
2. Do you have any difficulty using your arms?
3. Do you have any difficulty using your legs?
4. Do you have any difficulty using any other part of your body?
5. Do you need a mobility aid or prosthesis?
6. Do you have convulsions, involuntary movement, rigidity, or loss of consciousness (included are seizures associated with other MSI, such as cerebral palsy)?

If any of the answers are “yes”:

7. Has it lasted more than one month or is it permanent?

Following this screen, children underwent a standardized examination by an Orthopaedic clinical officer (OCO), paramedics trained to delivery primary care for orthopaedic conditions [18]. Examination included observation of activity, including mobility, hand function and ability to alter and hold positions. The OCO also undertook detailed histories and physical examinations for specific MSI conditions. Categorical data was collected for: i) the body region involved and details of the impairment, ii) the aetiology of impairment as far as could be assessed, iii) severity following ICF guidelines classified into mild, moderate and severe, iv) treatment required and received.The MSI subgroup was classified into congenital, traumatic, infective, neurological and acquired non-traumatic non-infective. Individual diagnoses were assigned using International Classification of Disease-10. These tools were designed for MSI surveys and have been tested in other LMICs [10].

Other clinicians screened children for hearing, visual, or intellectual impairment, and/or epilepsy, as appropriate, but these have been described in a pervious paper [15]. Demographic, socio-economic, school attendance and literacy data was also collected for each child.

### Statistical Analysis

Data was entered in Microsoft Access Database Management System. SPSS was used for analysis. In the subset of children who were referred by KIs but did not attend screening camps, it was assumed that prevalence and distribution of MSI were the same as those who did attend. Sensitivity analysis of this has been previously demonstrated [15]. Extrapolation to national level was performed using the number counted divided by 338,235 and multiplied by 1000 for prevalence per 1000. Data has been presented in numbers of patients and percentages.

## Results

### Study population

An estimated 15,000 children were referred to the screening camps as having a suspected impairment. This figure was based on data suggesting an average referral rate of 30 children per KI. A total of 7220 children were assessed in the 33 screening camps, with an average of 215 children per camp. This provides an estimated response rate of 48%.

### MSI demographics

**Fig. 1:**
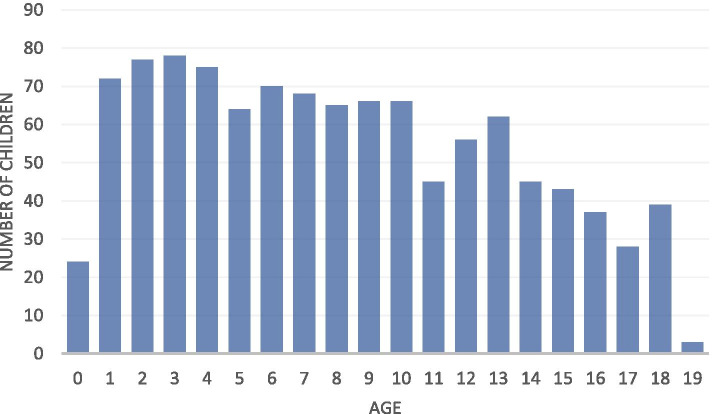
Number of children with MSI by age in the screened sample of 7220 children

Of the 7220 children screened a total of 1265 diagnoses of moderate/severe MSI were made in 1094 children (15.2%). Based on this, an estimated 2247 children had MSI within the districts studied, putting the estimated prevalence of MSI at 6.6/1000 (assuming the prevalence was the same in attenders and non-attenders of the camps). The number of MSI within the sampled peaked at 3 years old (134 children, 12.2%) (Figure 1).

Of the 1094 children with MSI, gender was recorded in 842, with 49% being female. Families were generally poor; 35% of the children with MSI had parents who were illiterate, 6.6% of parents could read and write but had no formal education, 44.1% had a primary school level education and 8.5% had a secondary school level education or beyond. The majority (88.8%) of children with MSI were in families with income less than 12,000 MK (Malawi Kwacha) per month (approx. 28 USD in 2013), 6.4% had an income of 12,000-30,000MK (28-71USD)/month, and 1.3% had incomes over 30,000 MK per month. Just over half (51.5%) of children with MSI came from Thyolo district.

### MSI Diagnosis and schooling

There were 1265 diagnoses of MSI made for 1094 children with MSI, categorised into four classifications. The number of MSI diagnoses were extrapolated to show a prevalence of 3.6/1000 diagnoses of neurological aetiology, 1.3/1000 congenital diagnoses, 0.891/1000 traumatic diagnoses and 1.19 acquired non traumatic diagnoses (Table 1).

**Table 1:** Numbers and prevalence estimates of children sampled with MSI

MSI impairment classification	Number assessed	Estimated Number^1^	Prevalence per 1000^2^	No. attending school
Neurological	591 (54%)	1228	3.630	170 (28.8%)
Congenital	215 (18.7)	447	1.321	123 (57.2%)
Trauma	145 (13.3%)	301	0.891	82 (56.6%)
Acquired Non-traumatic	194 (17.7%)	403	1.192	133 (68.6%)

^1^Estimated number assuming prevalence was similar in children who were referred but did not attend the survey

^2^Extrapolations using the total number of children in the villages (338,235)

^3^Number per million of all ages

At the time of questioning, 44.4% of children with MSI were currently enrolled at school. Most children with MSI attended public school but none attended schools known to have specific resources for children disabilities. Indeed only 0.24% of all children with disabilities attended disability inclusive schools with speciality resources. Among the children with MSI, school attendance was lowest for children with a neurological diagnosis (28.8%) and highest for those with Acquired non-traumatic diagnoses (68%) (Table 1).

### MSI aetiology

**Fig. 2:**
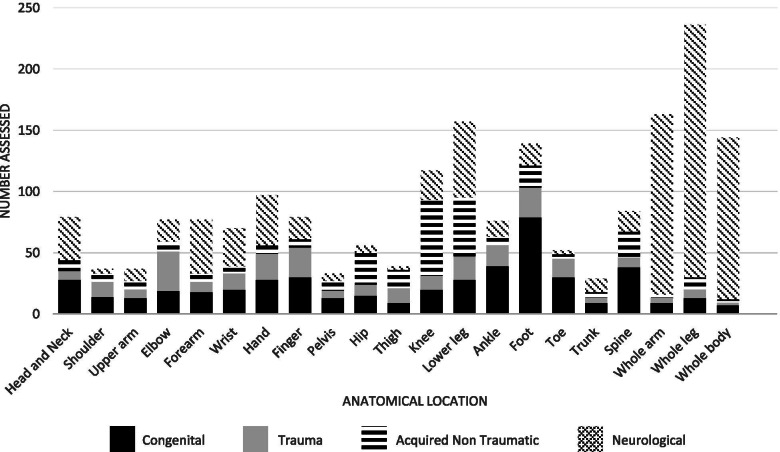
Graph showing anatomical location of impairment for the 4 different musculoskeletal impairment diagnoses

The aetiology was recorded 962 children; 24 children had multiple aetiologies (Table 2). The most common aetiologies were congenital MSI without family history (20.8%), cerebral malaria (15.1%), unknown (12.3%) and perinatal hypoxia (9.1%). The part of the body most commonly affected was were ankles, feet and toes for children with congenital MSI, thigh and knee for children with acquired non traumatic MSI and arm, leg or whole body impairment for children with neurological MSI (Figure 2).

**Table 2:** Aetiology of MSI

Aetiology	Number assessed		No. per 1000^1^
Family history	27	(2.9%)^2^	0.17
Congenital but no family history	226	(24.2%)	1.39
Perinatal hypoxia	103	(11.0%)	0.63
Trauma: Road traffic accident	6	(0.6%)	0.04
Trauma: Domestic violence	3	(0.3%)	0.02
Trauma: Other including accidents	68	(7.3%)	0.42
Infection: Osteomyelitis	23	(2.5%)	0.14
Infection: Cerebral Malaria	165	(17.7%)	1.02
Infection: Meningitis	20	(2.1%)	0.12
Infection: Other	62	(6.6%)	0.38
Developmental^3^	59	(6.3%)	0.36
Malnutrition	14	(1.5%)	0.09
Neoplasm	11	(1.2%)	0.07
Iatrogenic	5	(0.5%)	0.03
Unknown	141	(15.1%)	0.86

^1^Estimated assuming prevalence was similar in children who were referred but did not attend the survey and extrapolated using the total number of children in the villages (338,235)

^2^Percentages expressed as number counted of total number of children with MSI^3^Motor skills that are delayed at developmental milestones with no identifiable medical or neurological conditions

### MSI treatment recommended vs received

Of the 1094 children with MSI, most (52%) had not received a previous treatment. In addition to recommending physical therapy and surgery, other treatments recommended included mobility aids, Plaster of Paris, occupational therapy and wheelchairs. In total, 76% of children had not received the services recommended. This has been extrapolated to the population and is shown in Table 3.

**Table 3:** Treatment already received and recommended during survey

	Treatment previously received			Treatment Recommended from Survey		
Treatment	Number assessed		No. Per 1000^1^	Number assessed		No. per 1000^1^
None	570	(52.1%)^2^	3.51	154	(14.1%)	0.95
Medication	139	(12.7%)	0.86	72	(6.6%)	0.44
Plaster of Paris	66	(6.0%)	0.41	19	(1.7%)	0.12
Physical Therapy	221	(20.2%)	1.36	513	(46.9%)	3.16
Occupational therapy	11	(1.0%)	0.07	156	(14.3%)	0.96
Special seating	1	(0.1%)	0.01	4	(0.4%)	0.02
Mobility Aid	8	(0.7%)	0.05	26	(2.4%)	0.16
Tricycle	4	(0.4%)	0.02	16	(1.5%)	0.10
Appliance/Orthosis	5	(0.5%)	0.03	31	(2.8%)	0.19
Prosthesis	4	(0.4%)	0.02	9	(0.8%)	0.06
Wheelchair	15	(1.4%)	0.09	43	(3.9%)	0.26
Surgery	73	(6.7%)	0.45	192	(17.6%)	1.18
Traditional Medicine	4	(0.4%)	0.02	6	(0.5%)	0.04
Other	Na		Na	19	(1.8%)	0.12

^1^Estimated assuming prevalence was similar in children who were referred but did not attend the survey and extrapolated using the total number of children in the villages (338,235)

^2^Percentages expressed as number counted of total number of children with MSI

### Trauma Demographics

**Fig. 3:**
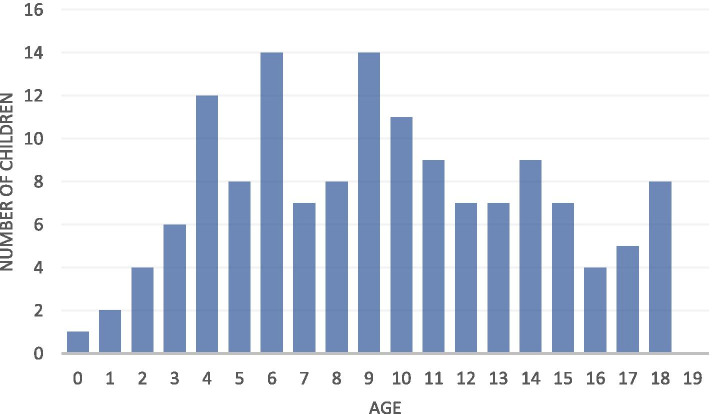
Number of children by age with traumatic MSI in the screened sample of 7220 children

A total of 145 children were found to have an MSI due to trauma (13.3% of all children with MSI). The number of children with traumatic MSI increased with age until the ages of 9-10. Most children reported the initial traumatic injury at ages 4 (28%) and 5 (25%) (Figure 3). Gender was recorded for 101 children, with 44 (30.3%) missing data. Of the 101, 47% were female.

### Trauma Breakdown

**Fig. 4:**
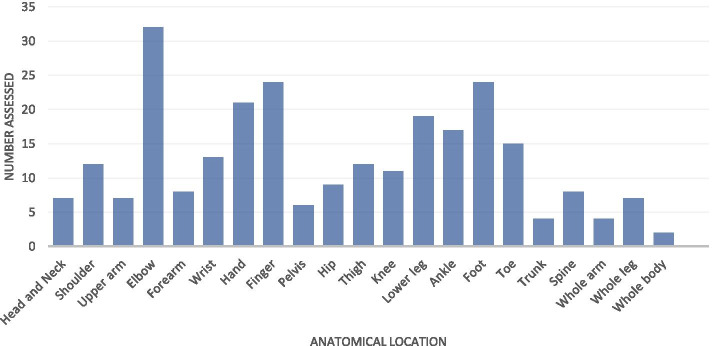
Graph showing anatomical location of impairment for the children with traumatic musculoskeletal impairment

Of the trauma aetiology, road traffic accidents (RTAs) accounted for 4% diagnoses of MSI, domestic violence for 2% diagnoses and other incidental accidents for 47%, while the remaining causes were “other” or no data recorded. The most common diagnosis of traumatic MSI was burn contracture (31%), followed by post traumatic joint stiffness (10.3%), fracture malunion (10.3%), peripheral nerve problems (10.4%) and amputation (11%). The extrapolations of these data are shown in Table 4. The most common body parts affected by traumatic MSI were elbow (22.1%), foot (16.6%) and finger joints (16.6%) (Fig. 4).

**Table 4:** Extrapolations of traumatic musculoskeletal impairment diagnoses

Diagnosis	Number Assessed		No. per 1000^1^
Burn Contracture	45	(4%)^2^	0.276
Post traumatic join stiffness	28	(2%)	0.172
Fracture malunion	15	(1%)	0.092
Head injury	1	(0.10%)	0.006
Recurrent/chronic dislocation	5	(0.40%)	0.031
Tendon/muscle problem	4	(0.40%)	0.024
peripheral nerve problem	15	(1%)	0.092
Amputation	16	(1%)	0.098
Cause not given	20	(2%)	0.123

^1^Estimated assuming prevalence was similar in children who were referred but did not attend the survey and extrapolated using the total number of children in the villages (338,235)

^2^Percentages expressed as number counted of total number of children with MSI

Of the 145 children with traumatic MSI, 67 children had received a total of 90 treatments. The most common treatment given was medication in 21 (14.5%) of children. Fourteen (9.7%) children had received plaster of Paris splintage, 11 (7.6%) had had physical therapy and 9 (6.2%) had received surgery. Seven children had reportedly received other treatment in the past but were not able to clarify what type. A total of 107 treatments were recommended for 84 children. Thirty children (25.9%) were recommended physical therapy and 26 (22.4%) children were referred for surgery. Nineteen children required 2 interventions and 5 children required 3 interventions. Thirty-nine children were followed up and 29 were referred for other services.

## Discussion

The KIM is a novel method to identify children with MSI impairments and estimate national prevalence. Extrapolating data to population level identifies national requirements and aids the planning of services and interventions [9].

This study has estimated a prevalence of paediatric MSI of 6.6/1000. This is similar to the data found in Bangladesh KIM survey (6.2/1000), which used both the KIM and a population-based survey to estimate prevalence [14], and to self-reported questionnaire from India and Cameroon, which showed difficulties in walking in 8/1000 [19]. It is not directly comparable to 2008 Malawi census data, which did not look specifically at MSI but reported impairment of walking in 16% of under 20s who had disabilities, giving a figure of 0.4/1000 for walking impairment [20]. Self-reporting is also likely to be affected by social and environmental factors and may be less reliable in terms of informing likely medical/rehabilitation needs than the clinical assessments of the KIM. The estimated prevalence from this study was lower than a survey in Rwanda, which showed a higher prevalence of MSI (26/1000 for ages 0-16) [21].

Neurological impairment was the most common diagnosis of MSI, with cerebral palsy accounting for almost a quarter of children. Many cases may be secondary to cerebral malaria [22]. Cerebral palsy is known to be the most common physical impairment worldwide, however it is a complex disease with a difficult diagnosis [23]. Due to the resource poor setting and differing aetiology in Africa, the term cerebral palsy may be inadvertently used to describe other neurological conditions [24]. Further research and education would help clarify this diagnosis. Our data shows that neurological MSI is likely to cause deficits in whole arms, legs or body. This indicates significant functional impact and a high requirement for neurological based disability services. Our results show that elbow injuries are the most common traumatic MSI, which is consistent with data from around the world [25, 26].

Understanding the aetiology of traumatic impairments is essential for prevention planning. We found that substantially fewer children (0.5%) reported MSI from RTAs compared to adult studies [21]. This may reflect the young age of the participants, who are less likely to be in or driving vehicles, and rural distribution of the survey, where there is less traffic. Very few children reported to have received their injury from domestic violence, but this may be due to underreporting. In contrast, a previous study in Malawi has shown the high proportion of interpersonal violence in adults, with most injuries from “assaults” [27]. Within the large number of children who described “other incidental accidents”, it is possible that some may have suffered abuse but did not feel empowered or safe enough to say. MSI due to infection, burn contractures and fracture malunions were also common. These are important findings, as impairment can be caused or exacerbated by delayed presentation [28]. Improving pre-hospital care and local service provision is key to reducing delay in presentation [9]. Though challenging in resource poor countries like Malawi, a national trauma registry can improve cost-efficiency of interventions , while community responders and transportation links can strengthen pre-hospital care response to trauma [9].

Only 44% of children with MSI from the survey were currently attending school, which is comparable to data from the Malawi census and other countries [3, 20, 29]. This is less than estimated school attendance of all children in Malawi (63%) [8]. Children who are excluded from school miss out on social participation, learning lifelong skills and gaining education to enable employment and social mobility [30]. There are conflicting reports on whether children with MSI are more or less likely to attend school than those with other disability types [2, 3]. Data from the same KIM showed attendance to be significantly higher for those with hearing impairments (91%) than those with MSI [15]. Attendance was particularly low in children with neurological aetiologies, potentially because of associated learning disabilities. Importantly, in our study no child with MSI attended a school with dedicated resources for children disabilities. This highlights the urgent need for accessible, disability-inclusive schools, where they are likely to have better outcomes [2]. Interventions such as increasing accessibility of classrooms, holistic education of both teachers and the community, and peer support groups for children can significantly improve learning achievements for children with disabilities [31].

The extrapolations from the MSI group show that about four per thousand children require physical therapy or occupational therapies. Our data shows that 60% of children had not received the requisite services. This exposes a huge unmet need for therapies and a high demand on existing services. This finding is supported by recent work showing that two thirds of the district hospitals in the Eastern, Southern and Central region of Malawi lacked rehabilitation services [32]. From our data only 6% had received surgery, while 17% of children with MSI required further surgery. Our results signify the need for further building of sustainable services to ensure long term benefit. A major objective is to increase the number of orthopaedic surgeons in the country. Training of surgeons should be concentrated in tertiary centres, where services can be increased efficiently [10]. In addition, the orthopaedic clinical officer program is a cost-effective and safe initiative that has already helped expand the capacity of orthopaedic care in Malawi [18, 33]. Other studies have shown most hospitals in Malawi lack essential infrastructure and resources to surgically manage trauma [32, 34]. Future surveys could assess the barriers to treatment in children with MSI, possibly using the Three Delays Model, which helps identify delays in seeking, reaching and receiving care [35]. Identifying which specific surgeries are needed would also be helpful to aid cost-effective and efficient scaling up of services.

Our study has several limitations. Just over half of the children referred to the screening camps did not attend. Our results rely on the assumption that the prevalence of MSI for non-attenders was the same as for those who attended. While a substudy on this cohort supports this assumption, our results may not be representative of the population [15]. It would be helpful to have more detailed questioning about economic status of families; almost all of the participants were in the lowest category of income. Further delineating wealth may be difficult in cash poor communities but other demographics could be included, such as occupation of parents. As previously stated, it is necessary to include more detailed questioning on aetiology of acquired disabilities. Finally our data is from 2012 and further developments in services may have occurred, but nevertheless this survey sets a baseline for measuring improvement. Further studies would be useful in assessing trends in both MSI itself, as well as treatment and services.

## Conclusion

This study has demonstrated a prevalence of MSI that is comparable to other LMICs and contributes to global epidemiological data on disability. It has highlighted the large number of children with MSI who are not attending school or do not have access to disability inclusive school resources. The study has also identified an enormous unmet requirement for treatment and the urgent need to scale up pre-hospital, surgical and rehabilitation care.

## Data Availability

The datasets generated and/or analysed during the current study are not publicly available due to privacy rules but are available from the corresponding author on reasonable request.

## Abbreviations

MSI: Musculoskeletal impairment
KIM: Key informant method
WHO: World Health Organisation
UN: United Nations
KI: Key Informants
ICF: International classification of functioning, disability and health
RTA: Road traffic accident
